# 2110. Enhancing Antibiotic Efficacy Against *Enterococcal faecium* with Bacteriophage and Antimicrobial Peptides

**DOI:** 10.1093/ofid/ofad500.1734

**Published:** 2023-11-27

**Authors:** Callan Bleick, Kyle Stamper, Sumaiya Mohib, Shaylyn Avery, Biswagit Biswas, Cesar A Arias, Michael J Rybak

**Affiliations:** Anti-Infective Research Laboratory, College of Pharmacy and Health Sciences, Wayne State University, Detroit, MI, Wayne State University School of Medicine, Department of Microbiology and Immunology, Detroit, MI, Detroit, Michigan; Wayne State University, Detroit, Michigan; 1. Anti-Infective Research Laboratory, College of Pharmacy and Health Sciences, Wayne State University, Detroit, MI, Detroit, Michigan; 1. Anti-Infective Research Laboratory, College of Pharmacy and Health Sciences, Wayne State University, Detroit, MI, Detroit, Michigan; 3. Naval Medical Research Command, Fort Detrick, MD, Fort Detrick, Maryland; Houston Methodist and Weill Cornell Medical College, Houston, TX; Eugene Applebaum College of Pharmacy and Health Sciences, Detroit, Michigan

## Abstract

**Background:**

The urgency to find new alternatives has increased as multidrug-resistant organisms enhance their defense mechanisms. In recent years, there has been a renewed interest in bacteriophages as potential alternatives or adjuncts to antibiotic therapy. However, there is limited information on the impact of bacteriophages with the innate immune system. Cathelicidin LL-37, a cationic peptide with antimicrobial properties, functions in coordination with the immune system to eradicate pathogens. We aim to evaluate how the survival of daptomycin (DAP) and ampicillin (AMP) resistant or non-susceptible *Enterococcus faecium* isolates are impacted by the interaction between bacteriophage, LL-37, and the addition of DAP/AMP.

**Methods:**

Two *E. faecium* clinical isolates were utilized: R497 and HOU503. Bacteriophages provided by the Naval Medical Research Command, NV- 503-01 and NV-497 were quantified and propagated to approximately 10^8^ PFU/mL. LL-37 was prepared at a final concentration of 640μM and diluted in RPMI intentionally to a suboptimal concentration of 0.5μM to adjust for a 70-80% survival rate. This was done to detect a synergistic interaction more easily with the addition of phage. Samples were incubated at 37 °C and plated in triplicate on brain heart infusion agar at 2h. After 24h of incubation, colonies were counted for and analyzed. Data was expressed as a mean percent survival with standard deviation. ANOVA with Tukey's HSD post-hoc test was used to determine the variations between each combination used.

**Results:**

The targeted bacterial survival rate against *E. faecium* isolates was attained by LL-37 at a concentration of 0.5μM. However, when R497 and HOU503 was combined with LL-37, bacteriophage, and DAP/AMP the percentage of bacterial survival was significantly (P< 0.05) lower than that of the growth control, LL-37, AMP/DAP, and bacteriophage alone.

**Conclusion:**

By conducting bacterial survival assays, we observed a notable enhancement in the elimination of multi-drug resistant *E. faecium* when LL-37, bacteriophage, and DAP/AMP were added. Examining this interaction over longer periods, both with and without varying antimicrobials, will yield more understanding regarding the potential involvement of bacteriophage in the innate immune system's response to fighting infections.

**Disclosures:**

**Michael J. Rybak, PharmD, PhD, MPH**, Abbvie, Merck, Paratek, Shionogi, Entasis, La Jolla, T2 Biosystems: Advisor/Consultant

